# The Influence of Different Sustainable Substrates on the Nutritional Value of *Tenebrio molitor* Larvae

**DOI:** 10.3390/foods13030365

**Published:** 2024-01-23

**Authors:** Agnė Jankauskienė, Dominykas Aleknavičius, Sandra Kiseliovienė, Šarūnas Antanaitis, Rimvydas Falkauskas, Marijona Šumskienė, Ignė Juknienė, Aistė Kabašinskienė

**Affiliations:** 1 Department of Food Safety and Quality, Lithuanian University of Health Sciences, Veterinary Academy, Tilzes St. 18, LT-47181 Kaunas, Lithuania; igne.jukniene@lsmu.lt; 2 Divaks, UAB, Vinco Kudirkos G. 22-12, LT-01113 Vilnius, Lithuania; dominykas@divaks.com; 3 Food Institute, Kaunas University of Technology, Radvilenu Pl. 19, LT-50254 Kaunas, Lithuania; sandra.kiselioviene@ktu.lt; 4 Agrochemical Research Laboratory, Analytical Department, Lithuanian Research Centre for Agriculture and Forestry, Instituto Al. 1, LT-58344 Akademija, Lithuania; sarunas.antanaitis@lammc.lt; 5 National Food and Veterinary Risk Assessment Institute, Tilzes St. 18, LT-47181 Kaunas, Lithuania; rimvydas.falkauskas@nmvrvi.lt; 6 Culinary Art and Wellness Laboratory, Alytus STEAM Open Access Center Food Technology, Faculty of Health Sciences and Engineering, Alytus College, Studentu St. 17, LT-62252 Alytus, Lithuania; marijona.sumskiene@akolegija.lt

**Keywords:** mealworms, by-products, trace element, chemical composition, sustainability

## Abstract

Every year, over 30% of food production is wasted. However, promoting a sustainable food supply not only fosters economic stability in agriculture and the food industry, but also safeguards precious natural resources and ensures universal food access and safety. Therefore, the aim of the study was to determine how specific growth conditions (utilizing by-products: sprouted potatoes (1), wheat bran (2), brewers’ spent grain (3), and a control sample with agar-agar gels (4)) affect the larvae of yellow mealworms (*Tenebrio molitor*). This includes their nutritional and energy value, consumer sensory profiling, and technological parameters of processing. The results have indicated that larvae reared on the substrate with wheat bran had the highest energy value, at 708.26 kcal. In larvae, the difference in protein content was not significant when changing the rearing conditions, and ranged between 48.54 and 59.18%. The larvae contained a significant content of fibers, with the highest amount detected in samples with brewers’ spent grain. The data indicate that glucose and arabinose were distinctive to larvae. Our study has also revealed a statistical difference in ash content between larvae and the substrate, with higher levels of nitrogen, copper, and zinc detected in the larvae compared to the substrate. We have found that the salt was naturally occurring in the substrates, with the brewers’ spent grain sample having the highest amount, at 1.83%. However, the control sample yielded the highest ratings, achieving a score of 7.30 for general smell acceptability. These findings emphasize the potential of utilizing various industrial and farm by-products as substrates for mealworms, transforming them into a sustainable and nutrient-rich food source. This contribution adds to the broader discourse on nutritional value and resource efficiency.

## 1. Introduction

The yellow mealworm (*Tenebrio molitor*) is a holometabolous insect with four life stages—egg, larva, pupa and adult—and is now distributed worldwide [1]. It is the first edible insect to be recognized as a novel food and has recently been assessed as safe for human consumption by the European Food Safety Authority (EFSA). The intended consumer population encompasses the entirety of the human populace [2].

The versatility of mealworms extends their applicability across various industries, highlighting their substantial contribution to circular production systems [2,3,4] and generating significant interest in this emerging research area [5].

Every year, a staggering USD 2.5 billion tons of food, which is equivalent to over one-third (30 percent) of all food produced, goes to waste [6]. Of great concern is the fact that only a small percentage of all food waste is composted; most of it goes straight to landfills and makes up a large portion of municipal waste [7,8]. Approximately one-third of the total waste is generated during the food production stage, including by-products such as brewers’ spent grain produced during beer production, wheat bran formed during the processing of grains, green and sprouted potatoes unsuitable for direct human consumption, etc. [9,10].

Additionally, mealworm larvae have a rich nutritional profile, with an average of 50% proteins and approx. 30% fat, the latter varying depending on the growing substrate [11,12]. Several studies have already been conducted confirming that mealworm larvae have a particularly balanced composition of amino acids and fatty acids, and they are rich in essential amino acids, monounsaturated and polyunsaturated fatty acids (oleic, linoleic, and linolenic). In addition, mealworm larvae contain certain amounts of minerals (copper, magnesium, iron, potassium, zinc and phosphorus) that sometimes even exceed the amount found in conventional meat [13,14]. It is known that the amounts of potassium, calcium, zinc and manganese found in the larvae exceed the amounts of these trace elements found in raw beef [14,15]. Studies show that the main carbohydrate component of mealworms is chitin–chitosan, which is obtained from the whole body of larvae [16,17]. Chitin positively affects consumers digestion, blood circulation and the immune system, and has anti-inflammatory properties [17,18]. Taking these findings into consideration, due to their rich nutritional value, it can be suggested that insects can also contribute to a more balanced diet for humans and animals.

However, there is a lack of research in this area. Only one study was found analyzing the influence of growing conditions on sugar content in mealworms, in which only a dry substrate of wheat bran (95% of the diet) was selected. In the mentioned study, a comparison was made by comparing the stages of larvae and pupae, emphasizing that a higher quantity of sugars was found in the larvae [19].

Reducing the adverse environmental impacts of human activities is achievable by exploring methods to utilize waste and selecting appropriate types of food. This approach aims to minimize the overall environmental footprint by as much as possible. Considering this, our study aimed to assess the impact of the substrate on the nutritional value of mealworm larvae. Our hypothesis posits that the substrate would not significantly affect the results, and that the nutritional value might surpass that of many commonly consumed European food products. The results of our analysis have shown that, by utilizing by-products from production/farms, we can obtain even higher nutritional values compared to the control group. The varied nutritional contents observed in the mealworms grown on various diverse by-products as a substrate carry practical implications. These findings suggested the potential for cultivating mealworms tailored to specific groups, health-related objectives, and various other purposes.

## 2. Materials and Methods

### 2.1. Larval Rearing Condition, Lyophilization and Coding

Yellow mealworm (*Tenebrio molitor*) larvae were reared under controlled conditions (temperature 27 ± 2 °C, humidity 60 ± 5%, lighting ≥ 1 h/day) at the Divaks company’s [20] insect research and development facility in Vilnius, Lithuania.

At the beginning of the experiment, the eggs were obtained from adult beetles of diverse age groups. The wheat flour from Kauno grūdai [21], Lithuania, was used as the egg-laying substrate, and adult beetles were provided with carrots as a source of moisture. The eggs were gathered at intervals of 3–4 days using a 0.5 mm sieve. Around 30,000 individuals, equivalent to 17 g of eggs, were deposited in 40 × 60 cm containers with 1.5 kg of dry feed, which was provided abundantly throughout the larval growth period, amounting to a total of 4 kg. Three experimental groups and a control group were formed. The samples were coded for the next tests (Appendix A). As the dry feed we used wheat bran from Fasma, Lithuania [22], with brewer’s yeast from Ekoproduktas, Lithuania [23] (corresponding substrates: SWYG, SWYP, SWYC), and dehydrated brewers’ spent grain from Eurokorma, Lithuania [24] (SBYC) with the same brewer’s yeast. Wet feed was given three times a week, including approximately 3.45 kg of carrots from Sanitex, Lithuania [25] (SWYC), 2.75 kg of green and sprouted potatoes from “Suvalkijos daržovės”, Lithuania (SWYP), and, in the control sample (agar-agar) (10 g/L) gels (Carl Roth, Germany, Darmstadt) [26] were given, totaling approximately 2.75 kg (SWYG). The ratio of dry feed to brewer’s yeast was 9:1 in all samples. The larvae were considered fully grown when the first pupae were found in the boxes. The fully grown larvae used for the study were raised for 56 days.

Following this timeframe, the larvae were sifted to remove waste and feed remnants using a 2 mm sieve. They then underwent a 24 h fasting period within the climate chamber before being re-sieved. Later, the larvae were frozen at −18 °C and transferred for subsequent analysis.

Mealworms and the perishable substrate components (green potatoes and carrots) underwent rapid freezing at −35 °C for 8 h using a Liebherr fast freezer (LGv 5010 MediLine). Freeze-drying was then carried out in a lyophilizer (Harvest Right, Salt Lake City, UT, USA) until reaching 80 °C. A pressure of 73 PA was applied during the freeze-drying process, which lasted a total of 72 h. Following this, the lyophilized larvae and substrate were processed milling using a laboratory-scale mill (Fritsch Mill Pulverisette 14, Darmstadt, Germany) at 6000 rpm.

### 2.2. Method for Determination of Protein Content

The tests were carried out in an accredited laboratory: National Food and Veterinary risk assessment institute, Kaunas, Lithuania [27]. The protein amount was determined by the Kjeldahl method according to the COMMISSION REGULATION (EC) No 152/2009 of 27 January 2009 laying down the methods of sampling and analysis for the official control of feed, Annex III, Part C [28].

### 2.3. Determination of Fat Content

The tests were carried out in an accredited laboratory: chemical science laboratory, food institute, Kaunas university of technology, Lithuania [29]. Fat was determined according to the standard: LST ISO 1443:2000, Meat and meat products—Determination of total fat content [30].

### 2.4. Determination of Carbohydrate Content

The formula used to calculate the amount of carbohydrates was [31]:

C = D.m. − (P + F + M.m.)(1)

where C—carbohydrate, g; D.m., P, F, M.m.—dry matter, protein, fat, mineral matter, g.

### 2.5. Determination of Energy Values

After determining the dry matter, mineral matter, moisture, fat and protein, the energy values were calculated according to the formula [32]:

E = 4 × P + 9 × F + 4 × C(2)

where E—energy value, kcal; P, F, C—amounts of proteins, fat and carbohydrates, g; 4, 9 and 4—energy value coefficients, kcal/g.

The energy value, as determined by laboratory tests, was calculated using the formula:

E = 4 × P + 9 × F + 4 [D.m. − (P + F + M.m.)](3)

where E—energy value, kcal; 4, 9 and 4—energy value coefficients, kcal/g; D.m., P, F, M.m.—dry matter, proteins, fat, mineral matter, g.

The errors were determined by comparing the amounts of proteins, fats and carbohydrates and the energy values determined by laboratory methods with their amounts calculated theoretically, according to the formulas:

(4) $$X_{P}=\frac{\left(P_{1}-{\;P}_{2}\right)\times 100}{P_{2}};\;X_{F}=\frac{\left(F_{1}-{\;F}_{2}\right)\times 100}{F_{2}};\;X_{C}=\frac{\left(C_{1\;}-{\;C}_{2}\right)\times 100}{C_{2}};{\;X}_{E}=\frac{\left(E_{1}-{\;E}_{2}\right)\times 100}{E_{2}};$$

where X_P_, X_F_, X_C_, X_E_—the errors for proteins, fats, carbohydrates and energy value (%); P_1_, F_1_, C_1_ and E_1_—the amounts of proteins, fats and carbohydrates (g), and the amount of energy; value (kcal, kJ) determined by laboratory methods; P_2_, F_2_, C_2_, E_2_—the amounts of proteins, fats and carbohydrates (g), and the energy values (kcal, kJ) determined by calculation.

### 2.6. Method for Determination of Sugars

The content of sugars was analyzed in an accredited laboratory: Kaunas University of Technology, Food Institute, Chemical Science Laboratory, Lithuania [29]. All reagents used were of analytical purity suitable for HPLC analysis. The water was of HPLC purity grade. The reagents used were as follows: Carrez I solution was prepared by dissolving 7.20 g of zinc sulphate (ZnSO_4_ × 7 H_2_O) in water and transferring the solution to a 100 cm^3^ volumetric flask and diluting to the mark. Carrez II solution was prepared by dissolving 3.60 g of potassium hexacyanoferrate (K_4_[Fe(CN)_6_] × 3 H_2_O) in water, transferring the solution to a 100 cm^3^ volumetric flask and diluting to the mark. The following substances were used for the test: sodium hydroxide solution, 0.1 M, and acetonitrile. Furthermore, to obtain accurate results, we used reagents with the specified concentrations (glucose ≥ 98%, fructose ≥ 99%, sucrose ≥ 99.9%, maltose ≥ 98%, lactose ≥ 98% arabinose ≥ 98%). An efficient liquid chromatography system (HPLC) equipped with an evaporative light scattering detector (HPLC system Shimadzu Corp., Kyoto, Japan) was used for the sugars analysis. An HPLC column with the following specifications was used: a column based on silica gel coated with polyamine, with a particle size of 5 µm, a pore size of 120 Å, an inner diameter of 4 mm, and an outer diameter of 250 mm (YMC Pack Polyamine II). Standards for the fructose, glucose, sucrose, maltose, lactose, and arabinose solutions were prepared by dissolving the necessary amount of substance in a 100 mL flask with HPLC water to get a concentration of 4 mg/mL of each standard substance. Preparation of the sample solution: the samples were weighed to between 4.00 and 5.00 g, approximately 50 mL of HPLC water was added, and the mixtures were thoroughly mixed. The samples were allowed to stand at 60 ± 2 °C in a water bath for 10–15 min. After removal from the water bath, the samples were cooled to room temperature, and 2.5 mL each of Carrez I and Carrez II solutions were added to each. The mixtures were quantitatively transferred to 100 mL volumetric flasks and diluted with HPLC water to the marks. The solutions were filtered through 0.2 µm-pore-size nylon membrane filters. The filtrates from each sample were analyzed using efficient liquid chromatography with an evaporative light scattering detector. To ensure test repeatability, three parallel analyses were conducted. Operating conditions: mobile phase flow rate—1.2 mL/min; injection volume—20 µL; column temperature—28 °C; isocratic elution, mobile phase consisting of acetonitrile and water (75:25 *v*/*v*). The identification of glucose, fructose, sucrose, maltose, lactose, and arabinose peaks in the sample was based on their retention times relative to the standard solutions. The results were calculated according to the formula:

(5) $$\mathrm{Amount}\;\mathrm{of}\;\mathrm{analyte}\;\left(g/100\;g\;\mathrm{product}\right)=\frac{S\cdot C_{\mathrm{St}}\cdot V_{1}\times 100}{S_{\mathrm{st}}\cdot 1000\cdot m}$$

where S—sample peak area; C_St_—concentration of the analyte standard mg/mL; V_1_—the volume at which the sample was prepared (100 mL); S_st_—peak area of the analyte standard; m—mass of the sample taken for analysis, in grams.

### 2.7. Determination of Salt Content

Three grams of finely ground larvae were weighed out, and then the prepared substrates were placed into a 200 mL beaker and 100 mL of distilled water was added. The samples were thoroughly mixed with a glass rod with a rubber tip (10 min) so that larger larvae or substrate particles would not remain and the salt would dissolve in the water. Then, the mixture was left to stand for 5 min.

A 15 mL pipette was taken from the settled liquid and titrated with 0.01 N silver nitrate solution using potassium chromate solution as an indicator. The amount of table sodium chloride (percentage) in the product under study was found according to the formula:

x = 0.0029 × v × 100 × 100/b × g(6)

where v—the amount of silver nitrate 0.05 N solution used for titration in mL; g—amount of ground larvae or substrates taken for the study in g; 0.05 N titer of silver nitrate solution.

### 2.8. Determination of Total Ash Content in Larvae and Substrate

The samples were prepared and analyzed as indicated in the study performed by Noyens et al. [33]. The results were recorded with an accuracy of 0.01%. Repeatability was applied to the method—the absolute difference between two separate test results obtained by one analyst using the same method when testing two test sub samples of the same sample in the same laboratory, with identical equipment, at the same time, must not exceed the value of r, calculated according to the formula:

r = 0.0990% + 0.00933 w.(7)

where w—an average of two results, expressed as a percentage.

### 2.9. Determination of Microelements

The tests were carried out in the accredited laboratory of the Analytical Department at the Agrochemical Research Laboratory, Lithuanian Research Centre for Agriculture and Forestry, in Kėdainiai district 58344, Lithuania [34].

The amounts of nitrogen, potassium, phosphorus, magnesium, calcium, selenium, iron, and copper were determined according to the European Standard: EN 15621:2017. For the animal feeding stuff, the methods of sampling and analysis involved the determination of calcium, sodium, phosphorus, magnesium, potassium, sulfur, iron, zinc, copper, manganese and cobalt after pressure digestion by ICP-AES [35].

The method used to determine the amounts of nitrogen and zinc was CEN/TS 16188:2012 for the sludge, whereas for the treated biowaste and soil, the determination of elements in aqua regia and nitric acid digests was carried out using the flame atomic absorption spectrometry method [36].

### 2.10. Method for Determining Moisture Content

The reference method was used to determine the moisture content: ISO 1442:1997, Meat and meat products [37].

Meanwhile, since the substrate was of plant origin, the method used for the test involved the determination of moisture content in cereals and cereal products via the reference method (ISO 712:2009) [38]. The samples were heated for 2 h in an oven at a temperature of 103 °C. The process of heating, cooling, and weighing was repeated according to the study until the difference between the results of two consecutive weighings (m_2_) after 1 h of heating was no greater than 0.1% of the sample mass.

### 2.11. Determination of Acidity (pH)

The pH of the samples was measured according to the standard method EN ISO 2917:2002 [39]. The pH in samples was evaluated with a pH meter (Inolab 3, Hanna Instruments, Milano, Italy). Before analysis, the pH meter was calibrated at two points, pH 4.01 and 7.00, using standard buffers (Sigma Aldrich, Saint Louis, MO, USA). The pH electrode was placed into the samples (larvae and substrate) of flour/water (1:1, *w*/*w*) for experiment preparation.

### 2.12. A Method for Determining Color Coordinates

The color coordinates of the mealworm were evaluated on the surface (Chromameter CR-400, Konica Minolta, Marunouchi, Japan). The parameters were measured in the reflection mode (L* (lightness), b* (yellowness) and a* (redness)) using a D65, 2° observer and an 8 mm aperture diameter.

### 2.13. Determination of Fiber Content

The amount of fiber was determined at the accredited Chemical Science Laboratory of the Food Institute at Kaunas University of Technology in Lithuania, according to the AOAC 985.29 method for total dietary fiber in foods, employing an enzymatic–gravimetric method [40].

### 2.14. The Method of Sensory Profiling

Sixty evaluators participated in the study; these were students of the Veterinary medicine program of the Veterinary Faculty of Lithuanian University of Health Sciences, trained to perform sensory tests. Before the study, the evaluators were familiarized with the course of the study, the properties to be evaluated, and the designations of the results. The samples were evaluated in a random order, and the temperature of the samples during the evaluation was about 20 °C. Each sample weighing about 5 g was submitted for evaluation in disposable containers. Between each sample, evaluators were given water to rinse their mouths so that the results of the analysis of one sample would not affect the results of the analysis of another sample.

The following sensory properties of mealworm larvae were evaluated and marked on a linear scale of 100 mm:

1. Overall color acceptability (from unacceptable to acceptable);
2. General acceptability of the smell (from unacceptable to acceptable);
3. The level of cohesion in the mouth (from low cohesion to high cohesion);
4. General taste acceptability (from unacceptable to acceptable);
5. Aftertaste in the mouth (from faint to bright);
6. Intensity of fat taste (from mild to intense);
7. Intensity of salty taste (from mild to intense);
8. Intensity of sweet taste (from mild to intense);
9. Intensity of bitter taste (from mild to intense).

The evaluation of sensory properties of all samples was performed three times.

### 2.15. Statistic

Data analysis was conducted using IBM SPSS Statistics 29.0.0.0 (241). Means and standard deviations of the studied characteristics of the compared groups were calculated. Differences between study groups were evaluated using ANOVA with Fisher’s LSD test. Differences are considered statistically significant when *p* < 0.05.

## 3. Results and Discussion

### 3.1. Energy Value, Carbohydrates, Fat, Proteins and Fiber

The highest energy value (Table 1) was found in larvae reared on a substrate with wheat bran (LWYC 708.26 kcal); however, there was no significant difference in energy value between larvae. The highest energy value of all substrates was found in sample SBYC (255.90 kcal), whereas the energy value in the larvae was 2.5–4.2 times higher than in the substrate. The average energy value in the larvae was significantly higher, at 688.7 kcal, compared to the substrate, which had an energy value of 196.8 kcal (*p* < 0.001).

The average carbohydrate content was statistically higher in the substrate (58.5%) compared to the detected amount in the larvae (9.4%) (*p* < 0.001). The substrate, particularly with wheat bran, exhibited the highest amount of carbohydrates (SWYC—63.09%). Compared to larvae, excluding the control (10.03%), the highest carbohydrate content was also found in the SWYC sample (9.57%).

The larvae that were cultivated on the substrate containing wheat bran (LWYC) exhibited the highest fat content. A significant difference was determined between the substrate (18.8%) and the amount of fat detected in the larvae (52.6%) (*p* < 0.001). Similar to the energy value and carbohydrate content, there was no statistically significant difference in the fat contents of mealworm larvae (*p* < 0.05). The LBYC sample had the lowest fat content, which was 15.32% less compared to the latter. However, the substrate of these larvae had the highest fat content. Noyens et al. reported an increase in fat content after adding potatoes to the diet. On the contrary, the studies of van Broekhoven et al. showed a decrease in fat after adding potatoes [12,33]. In our study, the SWYC sample had the greatest amount of carbohydrates.

Meanwhile, the larvae LBYC reared on beer production by-products displayed a substantial protein content of 59.18%. Additionally, the substrate used for these larvae also contained a high protein content of 23.25%, notably the highest among the tested substrates. Noyens and other researchers reared larvae on different substrates: on agar, as in our control sample, as well as on potato cuttings, vegetable mix, etc. [33]. The results show that the protein content (51.4%) in the aforementioned study was very similar to that in results found in our control sample. The highest content of proteins when using by-products was found in the sample with horticultural foliage (52.3%), while in our study the highest protein content was found in the LBYC sample with brewer’s by-products (59.18%). These results show that production/farm by-products do not make a significant difference, and can be successfully processed into protein-rich biomass using yellow mealworms. Thus, carbohydrates contain the largest part of the substrate compared to proteins and fat, and proteins contain the largest part of the larvae.

The LBYC sample had statistically the highest total and soluble fiber contents compared to other growing conditions (*p* < 0.001). According to Zheng and Mariod et al., if mealworms are reared on spoiled vegetables such as carrots, lettuce seeds, and Chinese leaves, the larvae can contain up to 6% fibers [41,42]. In our study, however, the highest amount of fibers varied from 5.03% to 8.07%.

### 3.2. Amount of Trace Elements and Ash

When comparing the trace element quantities in larvae cultivated on different substrates, it is evident that larvae grown on brewer’s by-products (LBYC) were the most proficient in this aspect (Table 2). In the LBYC sample, 8 elements out of 10 had the highest amount of trace elements, including their total amount.

According to Maret et al., about 25% of the world’s population is Zn-deficient due to poor nutrition [43]. The recommended amount of Zn per day is 7–10 mg according to age, and higher amounts are needed for pregnant women (about 11 mg/day) [44]. In our study, we observed particularly high Zn contents, reaching as much as 147.5 mg/kg in the LBYC sample. This implies that consuming less than 100 g of mealworms raised on brewer’s by-products would be sufficient to meet the daily Zn requirement, even for pregnant women.

Noyens et al. reported in their study that the larvae grown on a substrate containing fermented chicory roots exhibited the highest amount of Fe, at 45.19 mg/100 g [33]. In our study, using different by-products, we found the highest iron content in the LWYP sample, measured at 64 mg/kg. In contrast to the study, we did not detect a direct relationship between substrate and larval micronutrient content [33].

Significant differences in the total mineral content and trace elements are evident in the results presented in Table 3. Our study demonstrates a statistically significant difference (*p* < 0.001) in ash contents between larval and substrate ash. The amounts of nitrogen, copper and zinc were significantly higher in larvae compared to their respective substrate during growth (*p* < 0.001).

### 3.3. Amount of Dry Materials, Moisture, pH

Dried mealworms contain approximately 5% moisture, according to Mariod. Similar results were found in our study. The moisture content in samples varied between 3.48 and 7.43%, with the highest value observed in the LBYC sample (Table 4). This is presumed to be due to the fact that the lowest amount of fat was observed in this sample [41]. Our results support the statement of another author, Warner, who stated that higher protein content is associated with higher water holding capacity [45].

The lowest pH level was determined in the sample SBYC (5.15), while an equal pH was determined by Noyens et al. The researchers found that the pH of the vegetable mix (substrate) was about 5.1, and no significant differences in the pH values of the remaining substrates were detected [33]. The results do not show any direct relationship between substrate pH and larval pH. Products with a pH of approximately 7 are considered neutral, and according to the scientists Fenton and Huang, cancer develops in an acidic environment, while products with a pH of about 7 are the most beneficial for health [46].

### 3.4. Color Coordinates

The lyophilized and ground powder of mealworms had obviously different brightness values, which may have been caused by different substrates, since the conditions of cultivation and processing were analogous (Table 5). Statistically significant differences in lightness compared to other samples were found in the sample with LBYC (L* 70.87). In comparison with the results of other authors, the latter sample more closely resembled wheat flour than mealworms, according to color coordinates [47].

**Figure 1:**
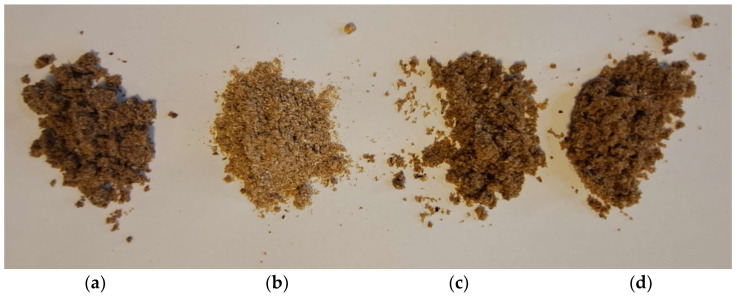
The photo shows color differences of lyophilized mealworms, grown under different rearing conditions: (**a**) LWYP sample (wheat bran + brewer’s yeast + srouted potatoes); (**b**) LBYC sample (brewers’ spent grain + brewer’s yeast + carrot); (**c**) LWYC sample (wheat bran + brewer’s yeast + carrot); (**d**) LWYG sample, control (wheat bran + brewer’s yeast + food agar gels).

Studies show that greater chroma (brighter, lighter color) is associated with a greater propensity for people to choose that product. It is likely that a person, using visual perception, will choose lighter products due to the association with freshness [48]. Both visual selection and color coordinate results indicate that the LBYC sample of mealworms was the most preferred product (Figure 1). One of the explanations why some larvae samples are particularly dark (LWYP) while the rest are lighter is the carrot-based component with β-carotene found in the substrate, as it is considered to be a natural dye [49].

### 3.5. Content of Salt

**Figure 2:**
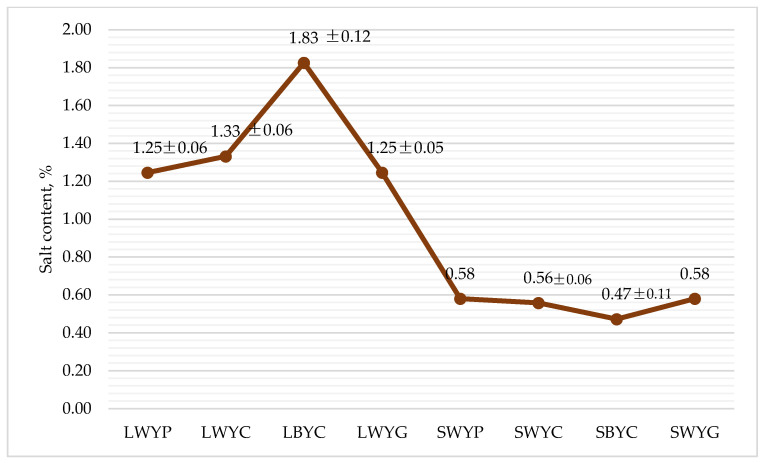
The content of salt in mealworms and substrate, %, average ± standard error, *n* = 3. LWYG—larvae, control (wheat bran + brewer’s yeast + food agar gels). LWYP—larvae (wheat bran + brewer’s yeast + green potatoes). LWYC—larvae (wheat bran + brewer’s yeast + carrot). LBYC—larvae (brewers’ spent grain + brewer’s yeast + carrot). SWYG—substrate, control (wheat bran + brewer’s yeast + food agar gels). SWYP—substrate (wheat bran + brewer’s yeast + green potatoes). SWYC—substrate (wheat bran + brewer’s yeast + carrot). SBYC—substrate (brewers’ spent grain + brewer’s yeast + carrot).

The salt was not intentionally added to the substrates, but it was detected as naturally occurring. The highest amount of salt was detected in the LBYC sample (1.83%) (Figure 2). Based on the obtained data, it can be assumed that the amount of salt in the substrate is inversely proportional to the detectable amount in mealworm larvae (*p* < 0.05). The salt content of mealworm larvae can vary depending on growing conditions and the larvae’s diet. *Tenebrio molitor* naturally contains a certain amount of minerals, including salt, which are crucial for their growth and development [50].

Anna Adámková et al. analyzed the optimization of conditions for better nutritional value using wheat bran and lentil meal in their study [51]. They showed that, respectively, about 0.1 and 6.7 mg of salt/100 g were found in the substrate of wheat bran and lentil.

### 3.6. Content of Sugar

A higher amount of fructose was detected in the substrate than in mealworms. No accumulation in larvae was observed. The highest amount of glucose detected in the sample LWYC was 3.9 g/100 g (Table 6). Sucrose was not accumulated by the larvae, although considerable amounts were detected in the substrates; for example, as much as 3.56 g/100 g was found in the SWYC sample. An analogous situation was found with maltose—although it was detected in the substrate (e.g., SWYC 2.76 g/100 g), the amount found in larvae was below the detection limit. Lactose was not detected in mealworms or in the substrates, suggesting that individuals with lactose intolerance can consume larvae produced on these by-products. The content of arabinose in almost all larvae and all substrate samples was below the detection limit, except for LWYP (4.43 g/100 g) and LWYC (5.94 g/100 g) samples.

To our knowledge, there is no study in which sugar content is measured in larvae grown under analogous conditions. However, in the study performed by Juan A. Morales-Ramos et al., the mealworms were raised on a substrate consisting 95% wheat bran and 5% food supplement (consisting of dry potatoes, dry egg white, soy protein and peanut oil) [52]. The results of the aforementioned study show that small amounts of fructose were formed in the larvae ((9.49 mg/g), corresponding to 0.0949 g/100 g. In our results, the highest amount of fructose was detected in the sample with wheat bran (LWYC)—3.9 g/100 g. So it is clear that our cultivation conditions led to a much higher amount of sugar in the larvae (41 times more) compared to the conditions used by A. Morales-Rama et al. [52].

The statistics show that when comparing the average accumulation of glucose (2.75 g/100 g) and arabinose (2.59 g/100 g) in larvae with the average amount in the substrate (0.89 g/100 g and 0 g/100 g, respectively), the sugars were specific to larvae (*p* < 0.001) (Table 7). Meanwhile, sucrose and maltose were not detected in the larvae, while their respective average amounts determined in the substrate were 2.38 g/100 g and 2.2 g/100 g, indicating that these sugars (*p* < 0.001) do not accumulate in larvae.

### 3.7. Sensory Profiling

Sensory evaluation is a method used to evaluate the sensations or perceptions induced by food in humans. This method is employed in the food industry as a research approach that assesses the quality of products and reflects consumer preferences [53].

Mealworms, like many other larvae or insects, can be evaluated very differently depending on cultural, ethical, and personal factors [19]. The consumption of insects, including mealworms, is a traditional practice in many parts of the world, where they are considered food and even delicacies [54]. However, in other countries, the eating of insects may be perceived as unusual or even unacceptable [55]. Petrescu-Mag et al. examined the attitudes of Romanian consumers towards mealworms as an ingredient in food. The results show that aversion to accidentally encountering insects in food does not affect the probability of eating *T. molitor*, and that after tasting this product from a young age, they include it in their diet without prejudice. One of the most important arguments for costumers to eat these larvae is their lower environmental impact [56].

**Figure 3:**
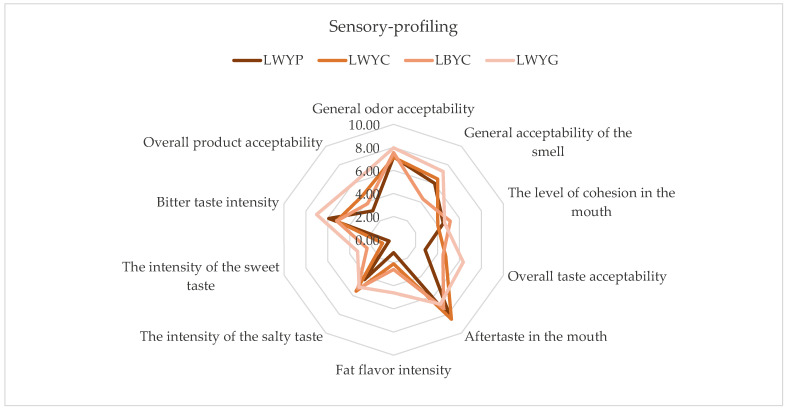
The sensory profiling of mealworms. LWYG—larvae, control (wheat bran + brewer’s yeast + food agar gels). LWYP—larvae (wheat bran + brewer’s yeast + green potatoes). LWYC—larvae (wheat bran + brewer’s yeast + carrot). LBYC—larvae (brewers’ spent grain + brewer’s yeast + carrot).

The profile analysis results show that the overall color acceptability among the samples was quite similar, and ranged from 7.14 to 7.96, indicating that the color aspect was relatively equally appreciated among all tested samples (Figure 3). The overall odor acceptability was also similar among the samples, and ranged from 6.03 to 7.30. This suggests that the tested samples were relatively well-received in terms of odor. The LWYG sample stands out with the highest overall color, odor, and taste acceptability compared to other larvae samples, and it is also quite fatty and salty. From the data, it can be inferred that the tested samples are relatively well-accepted in terms of odor and taste, although there are some differences in their taste intensity and mouthfeel. The LWYG sample stands out with the highest intensity of fat and salty flavors, while LWYC excels in terms of taste acceptability after consumption.

After performing a comparative analysis, statistically significant differences were found in the general acceptability of the smell. The smell of samples LWYC (6.49) and LWYG (7.30) was significantly different from that of LWYP (6.03) and LBYC (4.34) (*p* < 0.05) (Table 8). The overall taste acceptability of the LWYG sample (6.34) was significantly different from those of samples LWYP (2.87), LBYC (4.50), and LWYC (4.76). The overall product acceptability of the LWYG sample (5.99) was significantly different from those of samples LWYP (3.09), LBYC (3.84), and LWYC (4.64).

Kulma et al. utilized edible insects of various species and sizes in their study into descriptive sensory evaluation [57]. The assessment included chocolate cookies and white bread, incorporating insect meal in place of flour at 10%. In the referenced article, the highest overall pleasantness of taste (64–67%) was observed for *T. molitor*, while in our study, the taste was not incorporated into the dish, but pertains to the lyophilized larvae themselves, with the most similarity found in sample LWYG—6.34. However, both the overall acceptability and taste of the other samples (LWYP, LBYC, LWYC) were rated successively lower. Bartkowicz et al. confirmed the results of abovementioned study, finding that smaller insect particles are more acceptable [58]. Therefore, the comparison implies that larvae should be milled and integrated into dishes.

Wendin et al. considered that the addition of an antioxidant reduces bitterness, and the perceived taste depends on the size of the particles presented [59]. During our study, mealworm flour was used and no antioxidant was added, but the results have shown that bitterness (5.21–7.04) was certainly detected. No studies have been undertaken on the sensory profiling of lyophilized larvae themselves without other ingredients, but many researchers have tried to include mealworms in different food products already known to consumers, e.g., snack bars. The results of the study performed by Adamek et al. show that a positive consumer attitude towards energy bars with mealworms is registered, which indicates that Czech consumers accept edible insects in a suitable form as a new food and a possible part of their food purchase [60]. Roncolini et al. incorporated mealworms into bread products, and the results revealed that bread protein supplementation significantly affected bread texture, overall acceptability and crust color [61].

## 4. Conclusions

The study demonstrates that the substrate in which mealworms are cultivated does not exert a significant impact on the total nutrient content of the larvae. This suggests that mealworms can be efficiently transformed into a protein-rich biomass enriched with trace elements through the utilization of production and farm by-products. The energy value in the lyophilized larvae was 2.5–4.2 times higher than in the substrate. Meanwhile, the larvae exhibited a significantly higher fat content compared to the substrate. The amount of protein detected in larvae reared on beer production by-products (SBYC) was 59.18%, and the substrate of these larvae had the highest protein content (23.25%) as well. The LBYC sample demonstrated a statistically significantly higher content of total and soluble fiber among larvae grown with different feeding material mixtures (8.07%). Comparing the amounts of trace elements found in larvae grown on different substrates, it can be concluded that samples of LBYC had the highest contents of trace elements in this aspect (8 out of 10). The data indicate that glucose and arabinose sugars are distinctive to larvae. The salt naturally occurs in the substrates, with the highest amount detected in the LBYC sample (1.83 g/kg). The LWYG sample received the highest ratings, with 7.30 for general smell acceptability and 6.34 for overall taste acceptability. These findings are aligned with previous research, demonstrating the potential use of mealworms as a sustainable source of protein and other essential nutrients.

## Figures and Tables

**Table 1:** Energy value, carbohydrates, fat, proteins and fiber (insoluble and soluble) in mealworm larvae and feeding substrate, average ± standard error, *n* = 3.

Samples	Energy Value, Kcal	Carbohydrates, %	Fat, %	Proteins, %	Insoluble Fiber, %	Soluble Fiber, %	Total Content of Fiber, %
SWYG	183.27 ± 0.66 d	59.99 ± 2.88 a	4.16 ± 0.15 a	18.23 ± 0.02 d	-	-	-
SWYP	179.58 ± 0.46 ab	59.61 ± 3.14 a	4.13 ± 0.07 a	17.80 ± 0.00 a	-	-	-
SWYC	168.71 ± 0.07 b	63.09 ± 0.12 a	4.43 ± 0.02 b	16.11 ± 0.00 b	-	-	-
SBYC	255.90 ± 0.71 c	51.21 ± 3.57 b	7.77 ± 0.16 c	23.25 ± 0.10 c	-	-	-
LWYG (control)	689.27 ± 0.10 d	10.03 ± 0.51 b	32.54 ± 0.02 d	49.55 ± 0.05 d	5.0 ± −0.20	0.5 ± 0.0	5.5 ± 0.20
LWYP	701.64 ± 0.33 a	8.67 ± 0.18 a	30.78 ± 0.08 a	53.08 ± 0.01 a	4.53 ± 0.15	0.57 ± 0.05	5.1 ± 0.45
LWYC	708.26 ± 0.16 c	9.57 ± 0.48 ab	35.55 ± 0.03 b	48.54 ± 0.00 b	4.53 ± 0.2	0.57 ± 0.05	5.10 ± 0.25
LBYC	655.52 ± 0.04 c	9.34 ± 0.80 ab	20.23 ± 0.02 c	59.18 ± 0.00 c	6.47 ± 0.06	1.60 ± 0.29	8.07 ± 0.35

a, b, c, d—means marked with different letters in the row (in the groups larvae and substrate separately) differed significantly (*p* < 0.05, Fisher’s LSD criterion). LWYG—larvae, control (wheat bran + brewer’s yeast + food agar gels). LWYP—larvae (wheat bran + brewer’s yeast + green potatoes). LWYC—larvae (wheat bran + brewer’s yeast + carrot). LBYC—larvae (brewers’ spent grain + brewer’s yeast + carrot). SWYG—substrate, control (wheat bran + brewer’s yeast + food agar gels). SWYP—substrate (wheat bran + brewer’s yeast + green potatoes). SWYC—substrate (wheat bran + brewer’s yeast + carrot). SBYC—substrate (brewers’ spent grain + brewer’s yeast + carrot).

**Table 2:** Different trace elements and ash contents in mealworms and substrates, average ± standard error, *n* = 3.

	Units	LWYG	LWYP	LWYC	LBYC	SWYG	SWYP	SWYC	SBYC
Nitrogen	%	8.46 ± 0.01 d	8.73 ± 0.01 a	8.30 ± 0.01 b	10.24 ± 0.01 c	2.84 ± 0.01 d	3.02 ± 0.01 a	2.96 ± 0.01 b	3.82 ± 0.01 c
Potassium	%	0.99 ± 0.01 a	1.00 ± 0.01 a	0.91 ± 0.01 b	1.18 ± 0.01 c	1.00 ± 0.01 d	1.16 ± 0.01 a	1.25 ± 0.01 b	0.63 ± 0.01 c
Phosphorus	%	0.83 ± 0.01 c	0.72 ± 0.01 a	0.73 ± 0.01 a	1.03 ± 0.01 b	0.83 ± 0.01 d	0.95 ± 0.01 a	0.88 ± 0.01 b	0.73 ± 0.01 c
Magnesium	%	0.19 ± 0.01 b	0.15 ± 0.01 a	0.18 ± 0.01 b	0.23 ± 0.01 c	0.31 ± 0.01 d	0.29 ± 0.01 a	0.27 ± 0.01 b	0.22 ± 0.01 c
Calcium	%	0.034 ± 0.001 d	0.019 ± 0.001 a	0.031 ± 0.001 b	0.067 ± 0.001 c	0.230 ± 0.001 d	0.082 ± 0.001 a	0.220 ± 0.001 b	0.410 ± 0.001 c
Selenium	mg/kg	0.002 ± 0.0001 d	0.009 ± 0.0001 a	0.002 ± 0.0001 b	0.010 ± 0.0001 c	0.005 ± 0.0001 c	0.008 ± 0.0001 a	0.001 ± 0.0001 b	0.001 ± 0.0001 b
Iron	mg/kg	59.4 ± 0.2 d	64.1 ± 0.3 a	49.1 ± 0.4 b	47.1 ± 0.5 c	87.5 ± 0.11 d	110.0 ± 0.41 a	84.1 ± 0.61 b	200.5 ± 0.01 c
Copper	mg/kg	15.1 ± 0.04 d	15.6 ± 0.05 a	13.5 ± 0.2 b	13.7 ± 0.04 c	6.30 ± 0.12 a	6.15 ± 0.11 a	6.25 ± 0.14 a	8.05 ± 0.17 b
Zinc	mg/kg	133.5 ± 0.15 a	133.5 ± 0.13 a	128.0 ± 0.42 b	147.5 ± 0.61 c	57.3 ± 1.8 d	60.0 ± 1.22 a	54.3 ± 0.73 b	82.8 ± 1.53 c
Mineral content (ash)	%	3.08 ± 0.13 a	2.99 ± 0.14 ab	2.86 ± 0.13 b	3.81 ± 0.16 c	5.08 ± 0.13 b	3.98 ± 0.30 a	4.08 ± 0.02 a	5.01 ± 0.14 b

a, b, c, d—means marked with different letters in the row (in the groups larvae and substrate separately) differed significantly (*p* < 0.05, Fisher’s LSD criterion). LWYG—larvae, control (wheat bran + brewer’s yeast + food agar gels). LWYP—larvae (wheat bran + brewer’s yeast + green potatoes). LWYC—larvae (wheat bran + brewer’s yeast + carrot). LBYC—larvae (brewers’ spent grain + brewer’s yeast + carrot). SWYG—substrate, control (wheat bran + brewer’s yeast + food agar gels). SWYP—substrate (wheat bran + brewer’s yeast + green potatoes). SWYC—substrate (wheat bran + brewer’s yeast + carrot). SBYC—substrate (brewers’ spent grain + brewer’s yeast + carrot).

**Table 3:** Comparison of trace elements and ash contents in mealworms and substrate, average ± standard error, *n* = 3.

Material	Nitrogen, %	Magnesium, %	Calcium, %	Iron, mg/kg	Copper, mg/kg	Zinc, mg/kg	Mineral Content (Ash), %
Mealworms	8.93 ± 0.8 ***	0.19 ± 0.03 ***	0.04 ± 0.02 ***	54.9 ± 7.3 **	14.48 ± 0.94 ***	135.6 ± 7.6 ***	3.19 ± 0.40 ***
Substrate	3.16 ± 0.4	0.27 ± 0.04	0.24 ± 0.12	120.5 ± 49.3	6.69 ± 0.83	63.6 ± 11.8	4.54 ± 0.54

**—*p* < 0.01; ***—*p* < 0.001.

**Table 4:** Moisture content, dry materials and pH in mealworms and substrate, average ± standard error, *n* = 3.

Sample	LWYG	LWYP	LWYC	LBYC	SWYG	SWYP	SWYC	SBYC
Moisture content, %	4.80 ± 0.54 a	4.48 ± 0.17 ab	3.48 ± 0.50 b	7.43 ± 0.79 c	13.55 ± 2.84	14.48 ± 3.11	12.30 ± 0.12	12.77 ± 3.47
Dry materials, %	95.20 ± 0.54 a	95.52 ± 0.17 ab	96.52 ± 0.50 b	92.5 ± 0.79 c	86.45 ± 2.84	85.52 ± 3.11	87.70 ± 0.12	87.23 ± 3.47
pH	6.38 ± 0.04 ab	6.52 ± 0.05 a	6.50 ± 0.04 a	6.31 ± 0.25 b	6.38 ± 0.04 d	6.34 ± 0.02 a	6.26 ± 0.01 b	5.15 ± 0.03 c

a, b, c, d—means marked with different letters in the row (in the groups larvae and substrate separately) differed significantly (*p* < 0.05, Fisher’s LSD criterion). LWYG—larvae, control (wheat bran + brewer’s yeast + food agar gels). LWYP—larvae (wheat bran + brewer’s yeast + green potatoes). LWYC—larvae (wheat bran + brewer’s yeast + carrot). LBYC—larvae (brewers’ spent grain + brewer’s yeast + carrot). SWYG—substrate, control (wheat bran + brewer’s yeast + food agar gels). SWYP—substrate (wheat bran + brewer’s yeast + green potatoes). SWYC—substrate (wheat bran + brewer’s yeast + carrot). SBYC—substrate (brewers’ spent grain + brewer’s yeast + carrot).

**Table 5:** Color coordinates (L*—lightness, a*—redness, b*—yellowness) of mealworms and substrate, average ± standard error, *n* = 3.

	LWYG	LWYP	LWYC	LBYC	SWYG	SWYP	SWYC	SBYC
L*	55.4 ± 0.44 a	56.0 ± 1.57 a	56.77 ± 1.10 a	70.87 ± 0.40 b	70.27 ± 0.15 ab	71.73 ± 1.42 a	69.9 ± 0.79 b	61.4 ± 0.78 c
a*	5.6 ± 0.26 a	5.53 ± 0.21 a	4.90 ± 0.46 b	3.97 ± 0.15 c	6.17 ± 0.32 c	5.2 ± 0.20 a	7.87 ± 0.50 b	5.23 ± 0.25 a
b*	13.43 ± 0.25 a	14.23 ± 1.62 a	14.33 ± 1.19 a	18.1 ± 0.40 b	16.7 ± 0.40 a	16.03 ± 0.38 a	18.13 ± 0.57 b	14.9 ± 0.35 c

a, b, c—means marked with different letters in the row (in the groups larvae and substrate separately) differed significantly (*p* < 0.05, Fisher’s LSD criterion). LWYG—larvae, control (wheat bran + brewer’s yeast + food agar gels). LWYP—larvae (wheat bran + brewer’s yeast + green potatoes). LWYC—larvae (wheat bran + brewer’s yeast + carrot). LBYC—larvae (brewers’ spent grain + brewer’s yeast + carrot). SWYG—substrate, control (wheat bran + brewer’s yeast + food agar gels). SWYP—substrate (wheat bran + brewer’s yeast + green potatoes). SWYC—substrate (wheat bran + brewer’s yeast + carrot). SBYC—substrate (brewers’ spent grain + brewer’s yeast + carrot).

**Table 6:** The contents of different sugars (fructose, glucose, sucrose, lactose, arabinose, maltose) in mealworms and substrates (g/100 g), average ± standard error, *n* = 3.

Material	Fructose	Glucose	Sucrose	Lactose	Arabinose	Maltose
LWYG	0.37 ± 0.04 a	3 ± 0.09 c	<0.20	<0.20	<0.20 c	<0.20
LWYP	0.34 ± 0.04 a	2.16 ± 0.08 a	<0.20	<0.20	4.43 ± 1.41 a	<0.20
LWYC	0.37 ± 0.06 a	3.9 ± 0.25 b	<0.20	<0.20	5.94 ± 0.38 b	<0.20
LBYC	0.56 ± 0.15 b	1.95 ± 0.11 a	<0.20	<0.20	<0.20 c	<0.20
SWYG	0.49 ± 0.06 a	0.65 ± 0.08 a	1.25 ± 0.03 d	<0.20	<0.20	2.69 ± 0.03 d
SWYP	0.38 ± 0.03 a	0.71 ± 0.04 a	1.78 ± 0.03 a	<0.20	<0.20	2.19 ± 0.07 a
SWYC	0.9 ± 0.04 b	1.17 ± 0.02 b	3.56 ± 0.15 b	<0.20	<0.20	2.76 ± 0.10 b
SBYC	0.79 ± 0.13 b	0.96 ± 0.17 c	2.54 ± 0.12 c	<0.20	<0.20	1.31 ± 0.33 c

a, b, c, d—means marked with different letters in the row (in the groups larvae and substrate separately) differed significantly (*p* < 0.05, Fisher’s LSD criterion). LWYG—larvae, control (wheat bran + brewer’s yeast + food agar gels). LWYP—larvae (wheat bran + brewer’s yeast + green potatoes). LWYC—larvae (wheat bran + brewer’s yeast + carrot). LBYC—larvae (brewers’ spent grain + brewer’s yeast + carrot). SWYG—substrate, control (wheat bran + brewer’s yeast + food agar gels). SWYP—substrate (wheat bran + brewer’s yeast + green potatoes). SWYC—substrate (wheat bran + brewer’s yeast + carrot). SBYC—substrate (brewers’ spent grain + brewer’s yeast + carrot).

**Table 7:** Comparison of different sugars (fructose, glucose, sucrose, lactose, arabinose, maltose) in mealworms and substrate g/100 g, average ± standard error, *n* = 3.

Material	Fructose	Glucose	Sucrose	Arabinose	Maltose
Larva	0.41 ± 0.12 **	2.75 ± 0.81 ***	0 ± 0 ***	2.59 ± 2.83 **	0 ± 0 ***
Substrate	0.65 ± 0.24	0.89 ± 0.23	2.38 ± 0.89	0 ± 0	2.2 ± 0.64

**—*p* < 0.01; ***—*p* < 0.001.

**Table 8:** Sensory evaluation of mealworms, comparative analysis, average ± standard error, *n* = 3.

Sensory Characteristic	LWYP	LWYC	LBYC	LWYG
General odor acceptability	7.17 ± 0.74	7.14 ± 0.70	7.54 ± 2.11	7.96 ± 1.63
General acceptability of the smell	6.03 ± 2.08 ab	6.49 ± 2.28 ab	4.34 ± 2.53 a	7.3 ± 1.98 b
The level of cohesion in the mouth	4.53 ± 2.48	4.01 ± 2.21	5.16 ± 2.49	4.64 ± 2.23
Overall taste acceptability	2.87 ± 1.91 a	4.76 ± 2.90 ab	4.50 ± 2.14 ab	6.34 ± 1.96 b
Aftertaste in the mouth	8.45 ± 1	8.5 ± 1.43	7.36 ± 0.83	6.9 ± 2.39
Fat flavor intensity	1.14 ± 0.95 a	2.1 ± 1.49 a	2.6 ± 1.81 a	4.63 ± 2.57 b
The intensity of the salty taste	4.30 ± 2.87	5.53 ± 3.22	5.29 ± 2.45	5.10 ± 1.51
The intensity of the sweet taste	0.41 ± 0.41 a	1.01 ± 1 a	2.41 ± 1.33 b	3.29 ± 1.67 b
Bitter taste intensity	5.92 ± 1.72	5.21 ± 1.83	5.19 ± 2.86	7.04 ± 1.41
Overall product acceptability	3.09 ± 1.62 a	4.64 ± 2.41 ab	3.84 ± 1.36 a	5.99 ± 2.37 b

a, b—means marked with different letters in the row differed significantly (*p* < 0.05, Fisher’s LSD criterion). LWYG—larvae, control (wheat bran + brewer’s yeast + food agar gels). LWYP—larvae (wheat bran + brewer’s yeast + green potatoes). LWYC—larvae (wheat bran + brewer’s yeast + carrot). LBYC—larvae (brewers’ spent grain + brewer’s yeast + carrot).

**Figure A1:**
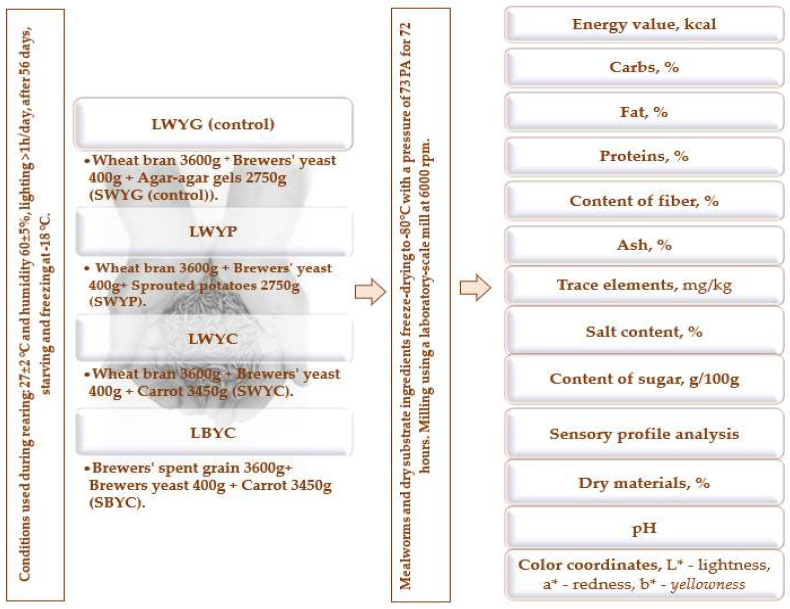
The whole process of the research. LWYG—larvae, control (wheat bran + brewer’s yeast + food agar gels). LWYP—larvae (wheat bran + brewer’s yeast + green potatoes). LWYC—larvae (wheat bran + brewer’s yeast + carrot). LBYC—larvae (brewers’ spent grain + brewer’s yeast + carrot). SWYG—substrate, control (wheat bran + brewer’s yeast + food agar gels). SWYP—substrate (wheat bran + brewer’s yeast + green potatoes). SWYC—substrate (wheat bran + brewer’s yeast + carrot). SBYC—substrate (brewers’ spent grain + brewer’s yeast + carrot).

## Data Availability

No new data were created.
